# COVID-19 containment management strategies in a nursing home

**DOI:** 10.31744/einstein_journal/2022AO6175

**Published:** 2022-03-07

**Authors:** Antonio Carlos Pereira Barretto, Danute Bareisys Salotto, João Francisco Lindenberg Schoueri, Jeane Mike Tsutsui, Celso Francisco Hernandes Granato, Marianna Barbosa Yamaguchi, Riciane de Carvalho, Nadir Zacarias, Antônio Sérgio Zafred Marcelino, Rogerio Rabelo, Wilson Jacob

**Affiliations:** 1 Universidade de São Paulo Hospital das Clínicas Faculdade de Medicina São Paulo SP Brazil Hospital das Clínicas, Faculdade de Medicina, Universidade de São Paulo, São Paulo, SP, Brazil.; 2 Fleury Medicina e Saúde São Paulo SP Brazil Fleury Medicina e Saúde, São Paulo, SP, Brazil.; 3 Casa Ondina Lobo São Paulo SP Brazil Casa Ondina Lobo, São Paulo, SP, Brazil.; 4 Rotary Clube de São Paulo São Paulo SP Brazil Rotary Clube de São Paulo, São Paulo, SP, Brazil.

**Keywords:** Coronavirus infections, COVID-19, SARS-CoV-2, Infection control, Communicable disease control, Nursing homes, Homes for the aged, Polymerase chain reaction, Carrier state

## Abstract

**Objective:**

To describe the first COVID-19 pandemic at *Casa Ondina Lobo*, a philanthropic nursing home in São Paulo city, and the containment measures against the pandemic that proved to be effective.

**Methods:**

Several preventive measures were taken before and during the pandemic, with emphasis on universal testing by reverse transcription polymerase chain reaction for COVID-19. All residents and employees were tested twice in a D9 period.

**Results:**

Among the 62 residents and 55 employees, in both testing, eight residents and nine employees tested positive for COVID-19. Of 22% of employees and 75% of residents evolved asymptomatic, emphasizing the importance of universal testing for the detection and isolation of these cases. A quarter of residents evolved without any symptoms, however, with COVID-19 signs, reinforcing the importance of monitoring vital signs. The second testing did not detect any new cases among residents, demonstrating the effectiveness of the containment measures, however, it found four new cases among employees. This emphasized their role in COVID-19 outbreaks in nursing homes. Only one patient died, a 12.5% lethality among those known to be infected and a 1.6% mortality in the total population of residents were seen.

**Conclusion:**

The adoption of appropriate containment measures enabled to contain an COVID-19 pandemic in studied nursing home. Universal reverse transcription polymerase chain reaction testing for COVID-19 has proved to be particularly important and effective.

## INTRODUCTION

The epidemic of coronavirus disease 2019 (COVID-19) disease has brought unprecedented global impacts since the Spanish flu epidemic.^(1,2)^

In recent decades, an increasing number of nursing homes has been observed. The Brazilian Society of Geriatrics and Gerontology in 2003 adopted the term long-term care facilities to define this institution, however, they are commonly known as homes for the elderly, or, perhaps, less appropriately, as asylums.^(3)^

Nursing home residents are vulnerable to COVID-19 infection due to their age, comorbidities, shared environment and dependence on others to perform daily activities.

Arons et al.^(4)^ published a report of an outbreak of COVID-19 in a healthcare facility in Washington (DC), in which residents were tested with reverse transcription polymerase chain reaction (RT-PCR). They found 63% of residents to be tested positive, 53% were asymptomatic, and mortality among them was 26%.^(4)^

Since the rate of asymptomatic patients is high, controlling an outbreak of COVID-19 by surveillance of signs and symptoms alone is not effective. Testing for the virus identifies asymptomatic patients, allowing for their isolation, which is helpful in containing these outbreaks.

In the published literature, the mortality rate in PICU residents in COVID-19 outbreaks ranges from 0 to 26% of residents, and the case fatality ranges from 0 to 33.7%.^(4,5)^

## OBJECTIVE

To evaluate the efficacy of reverse transcriptase polymerase chain reaction testing for COVID-19, and other preventive measures in the management of patients in a Long-Term Care Facility for the Elderly during COVID-19 outbreak.

## METHODS

*Casa Ondina Lobo* is a philanthropic and non-profit nursing home (ILPI - *Instituições de Longa Permanência para Idosos*), located in the city of São Paulo (SP), in the neighborhood of Santo Amaro. The capacity for 90 older individuals of both genders aged 60 years old or older, and who do not have personal resources or family support structure.^(6)^

The house structure is composed of two collective dormitory pavilions (16 residents in the men area and 17 residents in the women area) and two collective infirmary pavilions for more dependent residents (seven residents in the men area and 22 residents in the women area). Besides this, the facility includes a large common living area for the residents. The residents are cared for by a multidisciplinary team, and there are daily medical visits on weekdays.

Because of the establishment of the COVID-19 pandemic in the country, the administration of *Casa Ondina Lobo* has implemented several measures to prevent the outbreak of the disease in the environment since March 2020, in addition to containment strategies, if necessary, including new routines and the use of protective equipment.

In this context of concern about the elderly residents in nursing home, Rotary Brazil developed the *Corona Zero* Project,^(7,8)^ which main objective is to protect the elderly population of the country's nursing home, by performing RT-PCR tests for severe acute respiratory syndrome coronavirus 2 (SARS-CoV-2). This test is offered to all residents and employees of these institutions, even asymptomatic ones, in order to detect cases of the disease, carry out a coping protocol, and prevent an outbreak of the disease in the environment.

The universal testing was accepted and performed by all residents and employees of the nursing home in early May 2020, and again D9 later. By convention, the day of the first testing is referred to here as day D0, and the other subsequent days are counted in sequence. The second testing occurred on D8 and it was conducted with all residents and staff to assess the spread of infection and the effectiveness of prevention measures.

Until the date of the initial testing, no patients had shown typical symptoms of COVID-19. However, among the staff members, one of them, who worked at night shift assisting in the care of all the residents, reported fever that was confirmed by screening at admission, D8 before the D0. The staff member was immediately requested to do home quarantine.

The first test was performed in D0, by the *Fleury Medicina e Saúde* Group, by a properly trained and properly dressed employee of this institution (N95 mask, aprons, gloves, face shield, and goggles). After test reveal positive for residents and employees in D0 testing, positive patients were isolated in one ward and suspect patients (residents who were roommates of positive patients) were allocated separately in another isolation ward. Patients in these settings remained exclusively in these locations, not frequenting common areas, and a dedicated nursing staff member and a physician were instituted to evaluate them, reducing the risk of contamination of other residents. The employees who tested positive were removed and requested to do self-quarantine for D14, under monitoring of their general condition.

This study was approved by the Research Ethics Committee of *Hospital da*s *Clínicas* of the *Faculdade de Medicina* at the *Universidade de São Paulo* (HCFMUSP) declaration # 4.188.096, CAAE: 35092620.2.0000.0068. The Informed Consent Form was explained, and signatures of all involved or their legal guardians were collected.

## RT-PCR for COVID-19

The RT-PCR test was performed with material obtained from nasopharyngeal and oropharyngeal scrapings (one sample per person). Samples were immediately sent to the laboratory, where the material was subjected to extraction of the genetic material using magnetic beads. In the next step, the material was submitted to reverse transcription and amplification of the resulting DNA in the QuantStudio^®^ equipment (Thermo Fischer Scientific, Waltham, MA, USA). This analysis targeted the N gene of the COVID-19 virus. This step was performed in a one-step procedure, using the TaqMan^®^ Fast Virus enzyme (Thermo Fischer Scientific, Waltham, MA, USA). Specific primers and probes were also employed for amplification and detection of the viral genetic material (Integrated DNA Technologies Inc., Coralville, Iowa, USA). The detection of the resulting product was done by capturing the increasing FAM fluorescence signal throughout the amplification process. The control of the steps of this extraction, amplification and detection process was done in uniplex, detecting the presence of human genetic material in the same sample (RNAse P). Samples whose signal was positive with Ct up to 37 were considered positive. This process was developed and validated in the laboratory itself, following the criteria recommended by the College of American Pathologists (CAP).

### Evaluation of symptoms

Respiratory symptoms and significant changes in vital signs were observed and asked actively and daily by the nursing staff, once a day for independent residents and twice a day for dependents.

Symptoms were reported to the medical team and recorded in the medical record. Positive RT-PCR cases were re-evaluated daily by the medical team until D16, in an isolation environment.

The signs and symptoms that were particularly monitored were: respiratory distress, hypoxia (oxygen saturation by oximetry ≤92%), tachypnea (respiratory rate greater than 22 breaths per minute), signs of poor systemic perfusion (capillary refill time greater than 3 seconds, persistent hypothermia and hypotension), decreased level of consciousness, and signs of delirium.

### Preventive measures

Several prevention measures, D45 prior to the detection of positive cases, were adopted by the administration of *Casa Ondina Lobo*, following recommendations offered by health agencies.^(9–11)^ These recommendations included the following: distribution and guidance on the use of Personal Protective Equipment (PPE) for health professionals and employees (N95 masks, gloves, protective aprons, face protection equipment, and caps), suspension of visits by family members and volunteers. In addition, suspension of sales at the institution's thrift store, respect for the physical and social isolation of residents with a 1.5m distance between beds, exchange of sofas for individual chairs, and sings in the cafeteria tables with safe distances, agglomerations in living environments were avoided, restriction of the exit of the elderly, except in cases of urgency and emergency (consultation or hospital), anticipation of the trivalent vaccine against Influenza virus to residents and collaborators, maintenance of airy environments with natural ventilation, distribution of cloth masks to residents, availability of alcohol gel, guidance to residents and collaborators on proper hand hygiene, cough etiquette, and respiratory hygiene, screening of collaborators, by means of temperature measurement and active questioning about symptoms, performed daily upon arrival for work, change of clothes and shoes upon arrival at the nursing home, before starting activities, use of clothes and shoes exclusive to the institution, implementation of a single entrance and exit for collaborators and another for suppliers and entry and exit of materials, adequate sanitation of products that would enter the institution, adequate and more frequent cleaning of the environments of the house, reservation of a pavilion for cases of suspected patients and another for cases of confirmed patients, application of quaternary ammonium in all environments of the institution, before the outbreak and again during the outbreak,^(12,13)^ and availability of partnerships for psychological support to employees.

## RESULTS

Of the total of 62 residents, 39 were women (63%), with a mean age of 80.8 years, 69% were patients older than 80 years. The degree of dependency ranged from I for 43.54% of the residents, II for 30.64%, and III for 25.80%.

Among the residents, eight cases tested positive for COVID-19 on first testing (12.9%). Of these, six were women. One of the residents was hospitalized due to pulmonary focus sepsis unrelated to COVID-19 infection (investigation was performed, and this diagnostic hypothesis was ruled out).

Of the 55 employees, 44 were female, aged 18 to 66 years (mean age 46.8 years), and only seven employees were 60 years or older.

Five employees tested positive for COVID-19 on the first test (10%), four of them were nursing assistants and one an administrative assistant. None had severe symptoms.

After test positivity in residents and employees at testing in the D0, the positive patients were isolated. All prevention measures were maintained, and hospital referral of patients with signs of severity was performed.

The day after the RT-PCR results were received (D1), the patients with positive results were evaluated by the medical team. Two of these patients were symptomatic. One patient had a productive cough, with no dyspnea or other symptoms, a condition that remained stable until the D13 of evolution, when the symptoms improved. One of the patients was identified with low peripheral perfusion and hypothermia, in addition to reports that the patient was hypoactive. This patient, with moderate to advanced dementia, was referred for hospitalization, with hypotheses of cardiogenic shock and delirium, and the individual was admitted to the intensive care unit.

By the end of follow-up on D16, all other patients remained asymptomatic, but two had no symptoms.

One patient presented asymptomatic bradycardia (heart rate of 48bpm) in D4, and this individual was referred to the emergency room for evaluation, having been diagnosed with COVID-19 pneumonia by chest computerized tomography scan. The patient was hospitalized for D10, returning asymptomatic to *Casa Ondina Lobo* in D14.

A fourth patient had an isolated febrile peak (37.8°C) in D4, but the individual was asymptomatic.

In the second testing, no residents tested positive for COVID-19 that had not already been detected in the first testing. Due to the layoffs, five new employees were hired, three of whom were women, for a total of 55 employees. Four employees tested positive for COVID-19 in the second test, of noting is that they had not been detected in the first test. These group was composed by two nursing technicians, a laundry employee, and a cleaning employee. All experienced mild symptoms, such as fever, myalgia, ageusia, and cough. No individual required hospitalization.

Thus, in the two tests, eight residents were positive, with the need for hospitalization for two of them, and nine employees were positive, with no need for hospitalization.

Of all the infected patients, only one died. This patient was hospitalized, required care in the intensive care unit, and returned to *Casa Ondina Lobo* after D30, where remained for D7, and died after this period. However, it was unknown if the death was really due to COVID-19 and its complications, however, the death was considered related to complications due to COVID-19.

The lethality was 12.5% among patients diagnosed with COVID-19, and the mortality was 1.6% of the total population of residents of the house. There was no death among the staff.

Analyzing the rate of asymptomatic patients, four residents (50%) were totally asymptomatic and without signs. Furthermore, considering that two patients presented signs/symptoms only on the fifth day of follow-up, in the first D4 of the outbreak, 75% of the patients were asymptomatic and without signs. Regarding the employees, 22% were asymptomatic.

**Figure 1:**
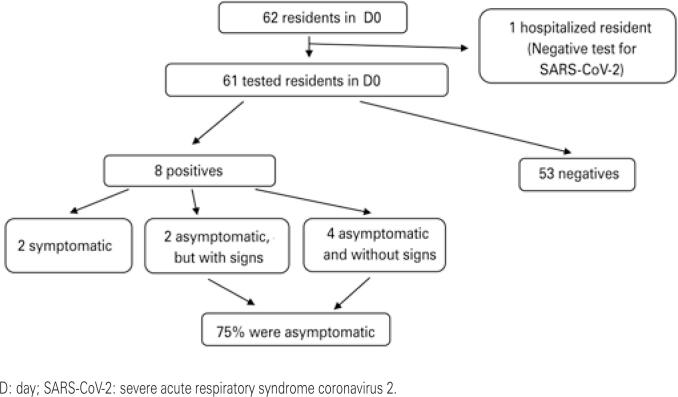
Results of reverse transcriptase polymerase chain reaction testing in residents on D0 and correlation with signs and symptoms

**Figure 2:**
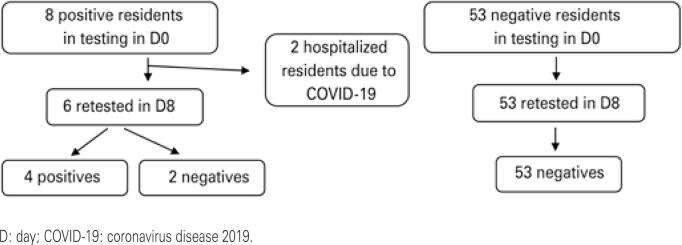
Results of reverse transcriptase polymerase chain reaction testing performed by residents in D8

Figures 1 and 2 summarize the results of the RT-PCR tests and the correlation with signs and symptoms in the residents.

## DISCUSSION

According to the literature, bradycardia can be found in patients infected with COVID-19.^(14,15)^

There were many cases of COVID-19 among long-term care facility residents and employees that would not have been detected without universal testing. This is consistent with several descriptions in the published literature of large numbers of asymptomatic cases among elderly patients, which contributes to the difficulties in containing these outbreaks.^(16)^

Two of the patients (25%) evolved asymptomatic, however, with signs of COVID-19 (fever and bradycardia, respectively), emphasizing the importance of monitoring the vital signs of elderly residents.

The social isolation and hygiene measures, along with the measure of physically isolating the infected patients in separate wards and keeping contaminated employees away. These were very effective in reducing new cases, and together with the measures already taken before the first test, played an important role in preventing new cases from occurring among the elderly in the second test.^(17)^

It is important to note that universal testing was already scheduled, and the first case of COVID-19 in our community occurred only D8 before the testing. Thus, the testing results generated a unique snapshot of the early stages of a COVID-19 outbreak in a long-term care facility.

In the second testing, D9 after the first, it was noteworthy that four new cases occurred among staff members, but none among the residents. This finding corroborated with the understanding in the literature that staff members play an important role in the entry and spread of the virus.^(18)^ These cases would have gone unnoticed if universal testing had not occurred, potentially leading to a new outbreak.^(16)^ Therefore, testing staff is a key point in outbreak prevention and containment.

Although all residents had comorbidities, only four patients (50%) had altered vital signs, and two (25%) were symptomatic. One patient had asymptomatic bradycardia, with no symptoms or changes in other vital signs, but on the chest computerized tomography scan, the patient had pulmonary involvement. This finding corroborates with other studies that have shown that the elderly often have asymptomatic or oligosymptomatic conditions.^(19)^ At the same time, mortality in this age group is disproportionately high.^(20)^

The COVID-19 virus eradicated in high concentrations from the nasal cavity even before symptoms are developed. This indicates that asymptomatic patients play an important role in the transmission of this disease. For this reason, the assessment based solely on surveillance of symptoms and vital signs may fail to detect infected patients and control outbreaks in these institutions.^(21)^

Currently, outbreak containment strategies have been diverse, depending on the association or country. What is known beyond doubt is that long-term care facilities populations are extremely vulnerable and require special care.^(22–24)^ In the present experience, measures such as personal hygiene, suspension of visits, and staff screening along with universal testing were effective in containing the pandemic.

This is one of the first descriptive papers addressing the issue related to long-term care facilities that reports the results and consequences of universal testing using RT-PCR during the COVID-19 pandemic in Brazil. This study also consistently demonstrated that pandemic was contained, therefore, indicating the best strategies for this purpose. The strategy of periodic RT-PCR testing for COVID-19 has been shown to be particularly effective in detecting asymptomatic elderly or staff, and, consequently, as a tool to prevent the spread of the pandemic, or the emergence of new pandemics at long-term care facilities.
